# The influence of oral health and psycho-social well-being on clinical outcomes in Behçet’s disease

**DOI:** 10.1007/s00296-018-4117-y

**Published:** 2018-08-27

**Authors:** Amal Senusi, Stephen Higgins, Farida Fortune

**Affiliations:** 1 Centre for Immunobiology and Regenerative Medicine, Institute of Dentistry, Barts and the London School of Medicine and Dentistry, Blizard Institute, Queen Mary University of London, 4 Newark Street, London, E1 2AT UK; 2 Behçet’s Centre of Excellence, Royal London Hospital, London, UK

**Keywords:** Oral ulcer severity score, Periodontal risk factors, Quality of life, Disease activity, Psycho-social well-being

## Abstract

This study was designed to investigate the association of oral ulceration and oral health factors, together with psycho-social well-being in Behçet’s disease (BD), and to clarify the importance of psycho-social support of patients in the overall management of BD. The study comprised of a cohort of 146 BD patients (mean age ± SD = 39.65 ± 13.20) and 20 recurrent aphthous stomatitis (RAS) patients (mean age ± SD = 42.32 ± 11.32). Oral ulcer severity score (OUSS), Behçet’s disease current activities form (BDCAF), hospital anxiety and depression scale (HADS), and the work and social adjustment scale (WSAS) were investigated. Oral health risk factors were also included. The analysis of variance, regression, and factor analysis were used to scrutinise the data. Almost 73% of patients were at high caries risk in BD and RAS groups. Thirty-nine percent of BD and forty percent of RAS had a score of BPE3 (probing depth 3.5–5.5 mm). Regression analysis revealed that OUSS and WSAS had a positive impact to increase the BDCAF score in BD patients (*β* = 0.395, *P* = 0.001; *β* = 0.240, *P* = 0.019), respectively. Dental health, periodontal health, anxiety, depression, and WSAS variables had strong loadings by factor analysis based on gender and at the time of present and absent of oral ulceration. The main oral ulcer characteristics that had significant influences on the total of oral health quality of life by 68.6% were: size, duration, ulcer-free period, and pain. The results highlighted the significant influence of oral ulceration, patients’ oral health, diet, and psycho-social well-being as multi-factorial causes on increasing disease activity in BD patients.

**Electronic supplementary material:**

The online version of this article (10.1007/s00296-018-4117-y) contains supplementary material, which is available to authorized users.

## Introduction

Behçet’s disease (BD) is a chronic, multi-system vasculitic disease. The aetiopathogenesis of BD remains unknown, and more recently, BD has been regarded as an auto-inflammatory condition. It is characterised with relapsing episodes of oro-genital ulcers accompanied by cutaneous lesions (pseudo folliculitis and erythema nodosum-like lesion), ocular symptoms (anterior uveitis, cataract, glaucoma, posterior segment involvement, and retinitis), arthropathy, vascular (arterial and venous vasculitis, and thrombosis), central nervous system (headache and meningoencephalitis), and gastrointestinal involvements (mucosal ulceration and perforation) [1, 2]. It may cause a significant morbidity such as blindness, loss of balance, deep venous thrombosis, and neurological problems [3]. In addition, fatigue and disturbance of sleep patterns are common in BD patients. The incidence is almost equal in males and females, although males often present with more severe symptoms [4]. Furthermore, the complex pattern of symptoms in adults with BD can have a negative effect on their psychological, physical, and social well-being, and can cause varying degrees of limitation of activity in everyday life [5].

### Oral ulceration and oral health in BD

The presence of painful recurrent oral ulceration is the first and the most common manifestation of BD which occurs in 80–100% of the patients, The ulcers may present in three different morphological patterns: minor ulcers, major ulcers, and herpetiform ulcers [6]. The cause of oral ulcers in BD is not fully understood. However, the oral ulceration in BD may be triggered by an abnormal immune response to an autoantigen. In addition, the clinical observations of BD suggest a potential role for antigens, especially organism, e.g., *Herpes Simplex* virus or *Streptococcus* Spp. in the pathogenesis. These infective agents may work through molecular mimicry, attracting inflammatory cells into the ulcerative area, and other cites of disease activity [7, 8]. Oral ulcers may also occur after local trauma causing during dental treatment [7, 9]. Other organisms such as *Candidiasis* are not uncommon in BD patients who are treated with immunosuppression [10]. Furthermore, the oral cytokines and immunomodulatory medication may also produce changes in the oral microflora in BD patients and causing increased pathogenicity [11, 12]. There is evidence that restoring the balance of the microbial community particularly in patients’ oral cavity was promoted increased healing of oral ulcers [13].

The previous literature suggests that, during the diseases activity, patients usually have (1) reduced oral hygiene, (2) high plaque accumulation, (3) decrease their tooth brushing activity, and (4) increase their intake of sugary drinks and soft foods which alters the oral pH and increased cytokines activity in saliva and gingival crevicular fluid and cause tooth decay and increase the number of extracted teeth due to multiple carious lesions [14, 15]. These factors are involved in the progression of the periodontal disease and can act to trigger their immune system and exacerbate the oral and systemic symptoms [16]. In addition, other studies have demonstrated the relationship of oral health and periodontal diseases in patients with BD compared to healthy participants [14, 17–19]. This suggests that improving the oral care of BD patients by plaque control methods: dental brushing, dental flossing, and regular dental examination may positively affect the course of the disease and prognosis [20] and may improve BD patients’ quality of life (QoL).

The frequent episodes of oral ulceration in BD patients subsequently cause mucosal changes including obliterative scarring. Our clinical observations have been that oral scars are seen in BD patients with major or herpetiform ulceration and particularly when the ulcers recurrent in the same location. Classically, BD patients who have ulcers in the oropharynx and oesophagus may develop severe scarring which may lead to oropharyngeal stenosis [21]. These scars can cause functional problems and may interfere with both swallowing and ultimately breathing. In addition, the changes have a negative impact on the patients’ QoL [22]. Besides, the oral health impact profile-14 (OHIP-14) questionnaire suggests that oral health problems in BD can alter the patients physical appearance leading to a reduction in QoL by impairing physical–social functioning and self-respect [23–25].

### Psycho neuro-immunology

Behavioural–neural–endocrine–immune system interactions suggest that psychological stress and negative emotions are related to an increase in the level of pro-inflammatory cytokines, e.g., interleukin (IL)-1β and IL-6, and predict illness and mortality [26, 27]. The increased levels of pro-inflammatory cytokines activate the hypothalamus and subsequently lead to “sickness behaviour”. This can disturb sleep, eating behaviour, and mood states, with the feelings of discomfort and low energy [28].

The link between long-term medical conditions, health, and well-being has been a focus of concern for service commissioners for several years [29]. Currently, there is interest in assessing the impact of oral health status on QoL and in assessing the impact of oral healthcare. Clinical oral health status measures have provided some insight into the physical, social, and psychological consequences of oral health.

Psychological stress and anxiety is known to have a significant role as a trigger or modifying factor in the onset and recurrence of recurrent aphthous stomatitis (RAS) [30–32], burning mouth syndrome (BMS) [33], and lichen planus (LP) lesions [34]. In response to stress, the alteration of hypothalamic–pituitary–adrenal axis (HPA) can lead to elevation of cortisol levels and a consequential activation of the immune system with an increased number of leukocytes at the sites of inflammation [35].

The focus of this research was to study the factors in the patients’ protocols that might impact on the BD patients’ outcomes including oral health, disease activity and oral health-related quality of life. These clinical outcomes might then assist in providing a more comprehensive approach to the patients’ overall management.

In this study, we have:

1. investigated the role of oral health factors, psycho-social well-being, oral ulcer severity, and disease activity in the BD patients based on the patients’ gender and oral ulceration (present/absent) episodes;
2. assessed the oral ulcer severity and OHQoL in RAS and BD by using the OUSS a new validated assessment tool;
3. explored the need of including oral health management and psycho-social support as a part of treatment guidelines of BD patients.

## Methods

The study was a year-long prospective study using a convenience sampling technique and was performed at Behçet’s centre (BD patients) and Oral Medicine Clinic (RAS patients) at the Royal London Hospital. BD and RAS participants were consented and included in this study which is a part of the City Research Ethical Committee (COREC) approved study “Immune-regulation at the mucosal barrier 14 November 2006” (P/03/122).

The eligibility criteria for inclusion patients were: for the BD, cohort was those patients diagnosed with BD according to International Study Group (ISG) 1990 criteria [36, 37]. The RAS cohort was those presenting with typical aphthous ulceration but no underlying haematinic deficiency or systemic disease. In both groups BD and RAS, all patients were between 18 and 65 years old. All females who were pregnant or lactating were excluded from both the BD and RAS group. The sample size was calculated using a specific formula that performed by Singh and Masuku [38]. The optimal number required in this study was 130 patients, and in this study, a large cohort of 264 BD patients had the oral health assessments: periodontal indices, periodontal risk factors, and dental caries index, OUSS, and OHQoL data, 157 females (mean age ± SD = 39.83 ± 13.42) and 107 males (mean age ± SD = 39.98 ± 11.95). In addition, 146 BD patients had all the oral health assessment plus to their psychological and social data (mean age ± SD = 39.65 ± 13.20). Twenty RAS patients (mean age ± SD = 42.32 ± 11.32) from the oral medicine clinic were also included.

### Outcome measures

#### Oral ulcer severity score (OUSS) form

The OUSS tool validated for BD and was provided to assess oral ulcer severity [39, 40]. The six OUSS characteristics: number, size, duration of ulcers, ulcer-free period, pain, and site of ulcers were recorded. The OUSS form used included evidence of scarring, to evaluate the severity of the oral ulceration in patients’ cohort; in addition, the effect of oral ulceration on normal life activity was investigated. The patients’ medication was also recorded for monitoring the efficacy of the treatment protocols for each patient. To complete the OUSS form, the ulcers’ characteristics were converted into numerical values, to assess the oral ulcers in BD and RAS patients for the last 4 weeks (Table S1).

#### Dental and periodontal examination

The dental and periodontal examination was carried out by the researcher on the day of clinical assessment of the patients. Decayed missing and filled teeth (DMFT) index was used for presenting the epidemiological data of the caries experience in BD and RAS cohort. When a carious lesion and a restoration are present, the tooth was recorded as a (D). When a tooth has been extracted due to caries, it was recorded as a (M). When a permanent or temporary filling was present, or when a filling was defective but not decayed, this was counted as a (F). The teeth not counted were: un-erupted teeth, congenitally missing teeth or supernumerary teeth, teeth removed, restored for reasons other than dental caries, third molars, and primary teeth retained in the permanent dentition.

Basic periodontal examination (BPE) was provided as a rapid-screening tool, and it was used to indicate the level of further examination needed and provide the basic guides on treatment need. BPE scores were given to each sextant, from 0 to 4, and * indicates dental furcation involvement [41] (Figure S1). World health organisation probe (WHO) was used for BPE. This has a “ball end” of 0.5 mm in diameter, and a black band from 3.5 to 5.5 mm. Light probing force was indicated (20–25 g). Sulcus bleeding index (BI) assessed the early sign of gingivitis [42], and plaque index (PI) assessed the thickness of plaque at the gingival area were applied for all the patients [43]. The patients were asked closed-ended questions answered with “Yes” or “No”; these included (1) patient’s gingival swelling or bleeding and family members swelling or bleeding; (2) smoking; (3) balanced diet; (4) medical problems, e.g., as diabetes, cardiovascular diseases and other comorbidities; (5) stress, and (6) oral hygiene habits (the frequency of brushing teeth, flossing, and cleaning tongue) were taken from all the patients; (7) plaque retention factors such as: supra-gingival calculus, sub-gingival calculus, and poor restoration margin were investigated by researcher.

#### Behçet’s disease current activity form (BDCAF)

The BDCAF form is a well-established tool for the assessment of BD activity in the clinic [44], which scores the history of clinical features on a 0–12 scale based on the number of symptoms which present over the preceding 4 weeks, prior to the day of assessment: these include headache, oral ulcer, genital ulcer, skin lesions, ocular symptoms, joint involvement, blood vessel involvement, and gastrointestinal and CNS complications.

#### Psychological and social well-being scales

Two measurement scales were used to evaluate the psychological and social well-being of patients; (1) hospital anxiety and depression scale (HADS), developed by Zigmond and Snaith [45], is a valid and convenient self-rating instrument for anxiety and depression in patients with somatic and psychological disorders. The questionnaire features seven questions relating to anxiety and seven relating to depression on 0–3 scales. The HADS is divided into four ranges: normal (0–7), mild (8–10), moderate (11–15), and severe (16–21). (2) The work and social adjustment scale (WSAS) is another valid and reliable self-report five-item questionnaire of functional impairment attributable to an identified problem or disorder on a 0–8 scale: 0 indicates no impairment at all and 8 indicates very severe impairment [46]. The range of the total score is 0–40, with lower scores indicating higher functioning. A WSAS score > 20 suggests at least moderately severe functional impairment. Scores between 10 and 20 indicate measurable functional impairment but less severe clinical symptomatology. The HADS and WSAS questionnaires were completed by patients on the day of their clinical assessment.

### Statistical analysis

The descriptive analysis was performed for mean and standard deviation values. The Pearson’s coefficient test was used to interrogate the data correlation. Regression, Kruskal–Wallis test (for non-parametric data), and analysis of variance (ANOVA) tests were implemented. The relationship between the variables was assessed by multivariate regression. Principle component analysis (PCA) was performed on the study scales with the number of factors predefined by eigenvalue ≥ 1.00 (eigenvalues are calculated and used in deciding how many factors to extract in the overall factor analysis). Varimax rotation was performed to obtain simpler factors. Rotation maximises the loading of each variable on one of the extracted factors whilst minimising the loading on all other factors. Interpretation of the PCA is based on finding which variables are most strongly correlated with each component; the correlation value ≥ 0.5 is deemed important.

The data were analysed using the SPSS Statistics software (version 20 for Windows; IBM Corporation, New York, USA). *P* values < 0.05 were accepted as significant.

## Results

### Dental and periodontal factors

Analysis of the DMFT and caries risk demonstrated that (106/146; 72.6%) of BD and (15/20; 75%) of RAS patients were at high caries risk. In addition, DMFT index components were used in form of three separated factors (D, M, and F factors) to be able to identify which factor/s had an impact on the total of DMFT index. Using the Kruskal–Wallis test to identify the DMF teeth in the BD group, resulted in a *P* value of < 0.001. However, there is no significant difference between DMF teeth in RAS patients *P* = 0.326.

BPE was carried out on BD and RAS patients. In the BD cohort, a total of (57/146; 39%) had BPE score 3, while (32/146; 22%) had BPE score of 4 and 7 BD patients had a BPE score of 4*(indicating probing depth > 5.5 mm plus a furcation involvement in the sextant). However, (41/146; 28%) patients had BPE score 2. Only three patients had BPE score 0. Most of RAS patients (8/18; 40%) had BPE score 3, and (5/18; 27%) had BPE score 4. Only two patients had BPE score 4*. The number of patients with BPE score 0 and 1 was [BD (3 and 6), no RAS had 0 and 1 BPE score], respectively (Figure S1).

(57/146; 39%) BD and (7/20; 35%) RAS patients gave a history of gingival problems, and 25 and 20% have only a family history of gingival problems (gingivitis and/or periodontal diseases), respectively. BD and RAS patients with plaque retention factors were (111/146; 76%) and (16/20; 80%) respectively. Most of our patients in both groups BD and RAS had never smoked. Only (43/146; 29.4%) BD and (3/20; 15%) RAS were smokers with mean number of cigarette consumption (2.5 ± 6.42/day) and (3.2 ± 7.22 day), respectively. Around 45% of our patients presented with only BD or RAS had underlying comorbidities. Six patients had BD and diabetes, 7 BD patients had cardiovascular disease, and 22 patients had BD with other medical conditions. Depression, anxiety, or other psychological and social problems were diagnosed in (54/146; 37%) BD and (7/20; 35%) in RAS patients. For most of our BD and RAS cohort, the diet was balanced and included all types of nutrition (according to the diet form and clinician decision). In terms of oral hygiene habits, most of the patients brushed their teeth at least twice/day and did irregular flossing and tongue brushing (Table S2).

The distribution of BDCAF scores (overall BDCAF score = 12) in BD patients was as follows: (6/146; 4%) patients had a score of 0, (9/146; 6%) patients had a score of 1, and (11/146; 7.5%) patients scored 2. (20/146; 13.7%) patients scored 3 and 5 each, (24/146; 16.5%) patients had a score of 4, (21/146; 14.4%) patients scored 6, (12/146; 8%), and (15/146; 10%) patients had a score of 7 and a score of 8 respectively. (7/146; 5%) patients scored 9, and only one patient scored 10.

Multivariate regression analysis and matrix correlation analysis demonstrated a moderate correlation between BPE with BI, PI, and with DMFT in BD [(*R* = 0.617; *P* < 0.001), (*R* = 0.408; *P* < 0.001), and (*R* = 0.549; *P* < 0.001)]. However, RAS group had a strong correlation between BPE with BI and with PI (*R* = 0.74; *P* < 0.001, *R* = 0.78; *P* < 0.001). There was a strong correlation between PI and BI in RAS (*R* = 0.76; *P* < 0.001) and moderate correlation in BD (*R* = 0.59; *P* < 0.001). There was a correlation between PI score and OUSS in both groups. The anxiety (mean ± SD = 9.16 ± 5), depression (mean ± SD = 6.79 ± 4.69), and the WSAS level (mean ± SD = 19.75 ± 9.65) were high in BD patients compared with RAS patients. The oral ulcer severity and psychological and social well-being were observed to be risk factors to increase the systemic activity in BD patients (*R*^2^ = 0.23, *P* < 0.001).

### The effect of gender on the oral health, and psychological and social well-being on BD

Principal component analysis (PCA) was used to analyse the effect of gender on oral health, disease activity, and psychological and social well-being. In these data, the largest eigenvalue, that is the eigenvalue of the first principal component, is (F1 = 3.61) in females group and (M1 = 4.22) in males group, and, therefore, explains 21.24 and 24.82% of the variation in the original data of females and males’ groups, respectively. Similarly, the eigenvalues of the second and third components were (15.33 and 10.24%) and (15.35 and 13.10%), respectively. The first three factors: F1, F2, and F3 together, therefore, account for 47.10% in women and 53.21% in men (M1, M2, and M3) of the variance in the original data. This information is summarized in Table 1 which also shows PC1 of BD females; under the heading, ‘F1’ has positive loadings on those variables [age, DMFT, number of filled teeth, BPE, and BI]. The PC2 under the heading ‘F2’ has positive loadings on the [BPE, BI, PI, and anxiety]. The third component under the heading ‘F3’ increased with increasing OUSS, BDCAF, depression, and WSAS, respectively. However, the PC1 in BD males under heading ‘M1’ has the same females’ variables; ‘F1’ except BI variable was less importance in males group, and the age range was important in males group in PC2 ‘M2’. The third component of BD males under the heading ‘M3’ soared with increasing the [anxiety, depression, and WSAS] and decreasing strongly with value of diet variable. This component ‘M3’ might be viewed that the healthy and balanced diet is a crucial factor in males BD group.

**Table 1:** Oral health, and psychological and social well-being of females and males BD patients

Principle component analysis results^a^						
	Component					
	F1	F2	F3	M1	M2	M3
Age range	**0.691**	− 0.093	0.114	0.124	**0.630**	− 0.105
DMFT index	**0.925**	0.216	− 0.009	**0.944**	0.209	0.006
Decayed teeth	− 0.139	− 0.043	0.094	0.254	0.055	0.271
Missing teeth	0.337	0.301	0.145	0.717	0.323	− 0.152
Filled teeth	**0.857**	0.080	− 0.114	**0.789**	0.053	− 0.065
BPE index	**0.530**	**0.636**	0.132	**0.521**	**0.633**	− 0.183
Bleeding index	**0.501**	**0.755**	− 0.119	0.334	**0.771**	− 0.010
Plaque index	0.002	**0.786**	0.141	− 0.005	**0.818**	0.115
OUSS	− 0.314	0.272	**0.608**	0.038	0.071	0.113
Smoking	0.061	0.078	− 0.376	0.183	0.307	− 0.203
Plaque retention factors	0.026	− 0.049	0.000	− 0.193	− 0.083	− 0.019
Medical factors	− 0.198	− 0.187	− 0.064	− 0.083	− 0.368	− 0.226
Diet	0.013	0.155	− 0.081	− 0.117	0.280	**− 0.588**
BDCAF	− 0.013	0.186	**0.569**	0.286	− 0.035	0.334
Anxiety	− 0.184	**0.674**	0.264	− 0.222	− 0.150	**0.829**
Depression	0.078	0.418	**0.593**	− 0.150	0.223	**0.773**
WSAS	0.133	− 0.060	**0.824**	0.116	− 0.037	**0.722**
Eigenvalues	3.61	2.61	1.75	4.22	2.61	2.22
Explained variance	21.24%	15.33%	10.24%	24.82%	15.35%	13.10%
Cumulative variance	21.53%	36.86%	47.10%	24.82%	40.17%	53.21%

Bold indicates factor loading ≥ 0.50 in the distribution of the oral health and psychological items

Extraction method: principal component analysis

Rotation method: varimax with kaiser normalization

*BPE* basic periodontal examination, *BDCAF* Behçet’s disease current activities form, *DMFT* decayed missing and filled teeth, *OUSS* oral ulcer severity score, *WSAS* the work and social adjustment scale (WSAS)

^a^Only cases for which Genders of BD = Female (F) and Male (M) were used in the analysis phase

It may be stated that, based on the DMFT correlation of 0.925 and 0.944 in the first components of females and males, respectively, these F1 and M1 are primarily a measure of the dental health. Whereas, F2 and M2 are measures for dental plaque on the correlation of 0.786 and 0.818. Finally, the prominent measure in F3 was WSAS and that in M3 was the anxiety. In summary, the key findings that DMFT and dental plaque were the main variables in both females and males. Moreover, F3 was primarily a measure for the work and social status. However, M3 was a measure for anxiety.

### The effect of oral ulceration on oral health, and psychological and social well-being in BD

The first three factors (PC1, PC2, and PC3) represent BD group with no oral ulceration (absence of oral ulceration), under the heading: AU1, AU2, and AU3 (eigenvalue ≥ 1.00) of accounted for 58.32% of the variance in the original data, while, in the group of oral ulceration (presence of oral ulceration), under the heading: PU1, PU2, and PU3, the cumulative variance value was 43.79%. The PC1 under heading ‘PU1’ of BD patients with oral ulceration was elevated (positive loading) with the following contributing variables (age, DMFT, number of filled and missing teeth, and BPE). The second component under the heading ‘PU2’ had positive loadings on (gender, BPE, BI, and PI). PC3 in BD group had strong positive values (BDCAF, anxiety, depression, and WSAS). However, the absence of oral ulceration in (PC1) of BD patients ‘AU1’ increased with fewer the number of decayed teeth and comorbidities such as diabetes and cardiovascular diseases and other medical problems, and ‘AU1’ increased with increasing the (age, number of filled teeth, and DMFT). The component ‘AU2’ increased with positive loading of the (BPE, BI, PI, and diet). The psychological and social well-being variables had positive loadings values with AU3 and PU3 components (Table 2). In summary, the first principle component of the absence of oral ulceration group (AU1) increased by decreasing the number of decayed teeth and increasing the number of filled teeth with the control being other medical conditions. AU2 is a primarily measure for dental plaque and diet. PU1 is a primarily measure for the DMFT index and PU2 for periodontal health. AU3 and PU3 are primarily a measure for the psychology and social well-being.

**Table 2:** Oral health, and psychological and social well-being of BD groups at the time of absence and presence of oral ulceration

Principle component analysis results^a^						
	Component					
	AU1	AU2	AU3	PU1	PU2	PU3
Age range	**0.707**	0.112	0.146	**0.605**	− 0.074	0.085
Gender	− 0.231	0.169	− 0.097	− 0.235	**0.613**	− 0.186
DMFT index	**0.563**	0.193	0.279	**0.877**	0.218	− 0.016
Decayed teeth	**− 0.728**	0.040	− 0.097	0.006	0.092	0.062
Missing teeth	− 0.053	0.261	**0.721**	**0.498**	0.211	0.060
Filling teeth	**0.829**	0.038	− 0.099	**0.811**	0.111	− 0.083
BPE index	0.358	**0.721**	0.174	**0.545**	**0.653**	0.120
Bleeding index	0.375	**0.771**	0.118	0.365	**0.796**	0.047
Plaque index	0.028	**0.866**	0.057	− 0.012	**0.794**	0.279
Smoking	0.234	0.243	− 0.108	0.133	0.206	− 0.287
Plaque retention factors	− 0.005	− 0.130	− 0.109	0.032	0.076	0.078
Medical factors	**− 0.656**	− 0.418	0.136	− 0.021	− 0.230	− 0.005
Diet	− 0.279	**0.785**	− 0.139	0.048	0.177	− 0.200
BDCAF	0.076	0.040	0.142	0.168	0.042	**0.534**
Anxiety	− 0.032	− 0.175	**0.784**	− 0.086	0.216	**0.771**
Depression	− 0.006	0.217	**0.842**	0.034	0.154	**0.825**
WSAS	0.404	− 0.154	**0.693**	0.069	− 0.132	**0.619**
Eigenvalues	4.96	2.59	2.40	3.56	2.19	1.70
Explained variance	29.16%	15.23%	13.94%	20.97%	12.88%	9.95%
Cumulative variance	29.16%	44.83%	58.32%	20.97%	33.84%	43.79%

Bold indicates factor loading ≥ 0.50 in the distribution of the oral health and psychological items

Extraction method: principal component analysis

Rotation method: varimax with kaiser normalization

*BPE* basic periodontal examination, *BDCAF* Behçet’s disease current activities form, *DMFT* Decayed missing and filled teeth, *OUSS* Oral ulcer severity score, *WSAS* the work and social adjustment scale (WSAS)

^a^Only cases for which OUSS in BD = Absence of oral ulcers (AU) and Presence (PU) of oral ulcers were used in the analysis phase

### Oral health in BD and RAS patients

Table 3 displays that PC1 under heading ‘BD1’ BD patients had positive loadings on [age, DMFT, BPE, and number of filled teeth]. The second principal component under the heading ‘BD2’ and the first principle component of RAS cohort under heading ‘RAS1’ had strong loadings on those variables (BPE, BI, and PI). ‘BD3’ with high values would tend to have a lot of plaque retention factors, in terms of supra-gingival calculus, sub-gingival calculus, and poor restoration margins, and ‘BD3’ component increased with increasing of medical problems. In summary, the RAS1 had a strong correlation with bleeding index of 0.955 that this principal component is primarily a measure of the periodontal health. RAS2 and RAS3 are responsible for dental and periodontal health. BD1 is primarily a measure of the dental and periodontal health. BD2 and BD3 were primary measures for the dental plaque and medical conditions, respectively.

**Table 3:** Assessment of oral health, and psychological and social well-being of RAS and BD patients

Principle component analysis results^a^						
	Component					
	RAS1	RAS2	RAS3	BD1	BD2	BD3
Gender	0.081	0.097	− 0.417	− 0.268	0.478	0.257
Age range	0.361	**0.615**	0.112	**0.652**	0.076	**0.301**
DMFT index	− 0.047	0.426	**0.878**	**0.907**	0.238	− 0.031
Decayed teeth	− 0.052	0.070	− 0.008	− 0.056	0.068	− 0.034
Missing teeth	− 0.090	**0.932**	0.140	**0.493**	0.295	0.174
Filling teeth	0.027	0.005	**0.952**	**0.803**	0.092	− 0.122
BPE index	**0.822**	0.450	− 0.027	**0.534**	**0.672**	− 0.128
Bleeding index	**0.955**	− 0.072	− 0.012	0.332	**0.783**	− 0.059
Plaque index	**0.875**	− 0.010	− 0.010	− 0.023	**0.856**	0.002
OUSS	− 0.053	0.016	0.081	− 0.197	0.102	0.106
Smoking	0.072	0.141	0.069	0.170	0.068	0.385
Plaque retention factors	− 0.351	− 0.107	− 0.037	0.097	0.022	**0.689**
Medical factors	0.225	− 0.445	− 0.009	− 0.078	− 0.174	**0.729**
Diet	− 0.145	− 0.095	− 0.120	0.039	0.005	− 0.012
Eigenvalues	4.07	2.65	1.90	3.54	2.29	1.80
Explained variance	27.14%	17.68%	12.61%	19.67%	12.70%	10.10%
Cumulative variance	27.14%	44.81%	57.42%	19.67%	32.37%	42.38%

Bold indicates factor loading ≥ 0.50 in the distribution of the oral health and psychological items

Extraction method: principal component analysis

Rotation method: Varimax with Kaiser normalization

^a^RAS and BD groups

### The association of OUSS with BDCAF score in BD patients

The analysis of regression test displayed that the study model (Dependent variable: BDCAF scores, and independent variable: overall OUSS) was significant (*R* = 0.395; *R*^2^ = 0.156; *P* = 0.001) (Table 4), and it indicated that the overall OUSS was responsible for more than 39.5% of the variance to increase the BDCAF score of BD patients.

**Table 4:** Regression analysis of OUSS with BDCAF score of BD patients

Model summary^a,b^	Unstandardized coefficients		Standardized coefficients	*P* value
	*B*	Std. error	Beta	
OUSS in BD	0.066	0.013	**0.395**	0.001*
*R* square value = **0.156**				
ANOVA test; *P* = 0.001*				

*Bold indicates* P* ≤ 0.05

^a^Predictor variable (independent variable): Overall OUSS of BD

^b^Dependent variable: BDCAF scores

### The association of psychology and social well-being with BDCAF score in BD patients

Table 5 indicates that the model of BDCAF scores as a dependent variable and the independent variables being anxiety scores, depression scores, and WSAS scores was associated significantly (*R* = 0.373; *R*^2^ = 0.139; *P* = 0.002). This also showed that the main influence to increase the BDCAF scores of BD patients was from the WSAS variable by 24% of the variance (*β* = 0.24; *P* = 0.019). In addition, there was no significant impact of medication on the BD patients’ psycho-social status.

**Table 5:** Regression analysis of psychology and social well-being with BDCAF score of BD patients

Model summary^a,b^	Unstandardized coefficients		Standardized coefficients	*P* value
	*B*	Std. error	Beta	
Anxiety scores in BD	0.266	0.286	0.103	0.353
Depression scores in BD	0.400	0.354	0.131	0.262
WSAS scores in BD patients	0.716	0.301	**0.240**	0.019*
*R* square value = **0.139**				
ANOVA test; *P* = 0.002*				

*Bold indicates *P* ≤ 0.05

^a^Predictors (independent variables): Anxiety, Depression, and Work and Social Adjustment Scale (WSAS) in BD patients

^b^Dependent Variable: BDCAF scores in BD patients

### The effect of oral ulcer characteristics on OHQoL in BD

The multivariate linear regression analysis (Table 6a) indicated that ulcer characteristics had an influence on the overall of OHQoL (*R*^2^: 0.686; *P* < 0.001). The beta values revealed that most of the contributions which make OHQoL worse were due to; ulcer pain, size of ulcers, ulcer-free period, and duration of ulceration (0.509; 0.250; 0.207; − 0.109: *P* < 0.001, 0.001, 0.001, and 0.027) respectively. Eating and talking were the major factors affected by high OUSS of BD: beta values were the same for both factors (0.364, *P* < 0.001: Table 6b).

**Table 6:** Model summary and coefficients of (a) oral ulcer characteristics and overall OHQoL in BD, and (b) OUSS and OHQoL factors in BD group

(a)				
Model summary^a,b^	Unstandardized coefficients		Standardized coefficients	*P* value
	*B*	Std. error	Beta	
Number of ulcers	− 0.067	0.139	− 0.023	0.629
Size of ulcers	0.784	0.152	**0.250**	0.001*
Ulcers duration	− 0.514	0.231	**− 0.109**	0.027*
Ulcer-free period	1.232	0.307	**0.207**	0.001*
Pain	1.460	0.165	**0.509**	0.001*
Site of ulcers	0.304	0.170	0.089	0.076
*R* square value = 0.686				
ANOVA test; *P* = 0.001*				

(b)				
Model summary^c,d^	Unstandardized coefficients		Standardized coefficients	*P* value
	*B*	Std. error	Beta	
Eating in BD	1.296	0.248	**0.364**	0.001*
Talking in BD	1.458	0.345	**0.364**	0.001*
Smiling in BD	0.514	0.313	0.122	0.102
*R* square value = 0.651				
ANOVA test; *P* = 0.001*				

*Bold indicates *P* ≤ 0.05

^a^Predictors variables: number of ulcers, size of ulcers, ulcers duration, ulcer- free period, pain, and site of ulcers

^b^Dependent variable: Overall OHQoL of BD

^c^Predictor variables: eating, talking, and smiling of BD patients

^d^Dependent variable: Overall OUSS in BD

## Discussion

It is widely accepted that oral health has a role to initiate or aggravate BD symptoms. This study was carried out to investigate the oral health, medical factors, psychology, and social well-being that might be associated with oral ulcer severity and disease activity.

The results showed that oral health determined by plaque retention factors, periodontal diseases, and the dental health (DMFT, particularly the number of filled teeth) were observed to be increased in both genders of BD and RAS patients. The increasing in number of filled teeth indicates that dental caries rates were higher in BD patients which also suggests that our patients’ cohorts were regularly attending their dental clinics where their lesions were treated. In the BD male group, the diet was an important variable and that might have an impact on the frequency and severity of oral ulceration comparing with BD female. The reasons might include the observation that the gastric intestinal tract (GIT) of males and females react differently to diet. This was apparent even when the nutritional intake is identical. In addition, sex hormones effect on the GIT microbiota composition, consequently, can trigger the immune system and cause inflammation [47, 48]. It has been well documented that women and men have different attitudes and knowledge about nutrition and what constitutes a balanced diet, and the requirements of a healthy diet. More studies and controlled trials are needed to establish whether the gender and diet directly affect BD and how they affect the disease presentation.

In addition, the oral health and comorbidity found in the BD and RAS groups were synergistically contributing to the high positive loading values to activate the disease. BD patients at the time of oral ulceration had those represented factors: PU1 (age, DMFT, number of filled teeth, and BPE), PU2 (BPE, PI, BI, and gender) and PU3 (BDCAF, anxiety, depression, and WSAS) were actively influencing the oral ulceration and the disease manifestations. In BD patients with active oral ulcers: the pain, size of ulcers, and duration of ulcers are the main ulcer characteristics that have an impact on OHQoL. Consequently, eating and talking are the main factors that were impaired by oral ulceration. Therefore, in our Behçet’s centre, enhanced guidelines have been developed which includes topical mouth ulceration treatment (steroid mouthwash and Barts mouthwash^↑^), and oral hygiene instructions are used to treat and educate our patients to follow preventive strategies, also to minimize the frequency of their disease activity and that will might lead to decrease the dose of their systemic medications (Table S3).

The strong positive loading values of psychological and social well-being variables (anxiety, depression, and WSAS) at the time of either the presences or absence of oral ulceration in the BD cohort were almost the same. This suggests that BD patients were generally at risk of mood and anxiety-related difficulties as well as experiencing impairment in their social functioning rather as a function of the presence of oral ulceration. This is not entirely surprising given that the multiple symptoms BD patients often experience together with the potential impact of a remitting–relapsing condition on their social functioning.

Psychological status has previously been identified at a higher prevalence in BD patients [49] and stress is associated with relapsing and remittance of symptoms in BD [50]. In addition, our findings showed that the WSAS status in BD patients had a positive impact on increasing the BDCAF scores. However, anxiety and depression did not show any association or impact on the BDCAF scores. These findings might guide us to use the patients’ health questionnaire 4 (PHQ-4), patients’ health questionnaire 9 (PHQ-9), and/or generalised anxiety disorder assessment (GAD-7) in any future studies instead of the hospital anxiety and depression scale (HADS), which was used in this research, as this might more accurately enable an understanding of anxiety and depression outside of the hospital setting, i.e., at home and in normal day-to-day life experience.

Interestingly, mood impacts on oral health through psychoneuroimmunological and behavioural processes. Behaviourally, low mood impacts on patients’ volition to undertake everyday living and personal care activities. In oral health, depression and stress are seen to reduce pro-health behaviours such as tooth brushing, flossing, and help-seeking in people with long-term medical conditions [51], and might increase pro-disease behaviours such as smoking and drinking alcohol [52]. Psychoneuroimmunological processes influence mucosal wound healing directly [53] and psycho-social stress is associated with BD relapse generally [50]. QoL and social functioning are affected by BD disease [54] and depression [55] independently though there remains a little clarity around the interaction of BD and mood on social functioning in BD patients. This combined effect may be additive and form a feedback mechanism, whereby impaired social functioning exacerbates depression and possibly relapsing BD symptoms.

This study has showed the importance of oral health factors and diet in both BD and RAS patients. In addition, factor analysis demonstrated that oral health factors, medical factors, and psycho-social well-being had significant impacts on BD activity. It also highlights the validity and usefulness of OUSS as a severity monitoring tool for BD oral ulcers and it is recommended that it may also be useful in other Behçet’s centres. In addition, including the oral hygiene instructions and using therapy mouthwashes are also highly recommended to be included in the first-line management  of oral ulcers in BD patients. In addition, having a healthy balanced diet should be considered as a part of the overall management of BD patients. The PHQ-4, PHQ-9, and/or GAD-7 are important to be used in any BD future studies instead of HADS, which was used in this research and the previous studies. Stress and patients’ inability to cope with the social activities and emotions might have a significant impact on BD patients. This study strongly suggests that psychological support is needed for BD patients. The Behçet’s centre at Barts Health NHS Trust provides such support. Finally, this is an area of clear interest in BD patients and merits further investigation.

## Electronic supplementary material

Below is the link to the electronic supplementary material.

Supplementary material 1 (PDF 235 KB)
