# Supplementary material for: The influence of oral health and psycho-social well-being on clinical outcomes in Behçet’s disease
Source: Rheumatol Int. 2018 Aug 27;38(10):1873–83. doi: 10.1007/s00296-018-4117-y (PMC6132727; doi:10.1007/s00296-018-4117-y)

Table

Table S1: OUSS form which includes the six ulcer characteristics and elements of oral function

Oral Ulcer Severity Score (OUSS)

In the last 4 weeks

Name:

Diagnosis:

Date of birth:

First visit to the department: Yes ☐ No ☐

Hospital Number:

Patient on medication for ulcers: Yes ☐ No ☐

Date:

Name of medication:

Clinician:

Duration on medication:

	Ulcer Characteristics	Score	Description of USS
Average **Number** of ulcers			Score = average number of ulcers in a crop<br>Maximum score = 20
Average **size** of ulcers (in mm)			Score = average size of ulcers in mm<br>Maximum score = 20
Average **Duration** of ulcers (in weeks)			Score = number of ½ weeks<br>i.e. Half week (3 days) scores 1, one and a half week (10 days) scores 3.<br>Maximum score = 9
**Ulcer-free period** (in weeks)			Score = 4 minus the average ulcer-free period in weeks<br>Maximum score = 4 (never free from ulcers
**Pain** as perceived by the patient (on a scale of 0-10)	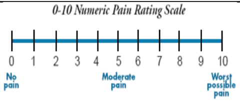		1 for slight discomfort when ulcers are present<br>10 for excruciating ulcers interfering with eating, talking or talking<br>Maximum score = 10
Mucosal **site**	**Group 1**<br>Labial mucosa<br>Buccal mucosa<br>Buccal sulcus<br>Soft palate<br>Ventral of tongue<br>Lateral of tongue<br>Floor of mouth<br>**Group 2**<br>Hard palate<br>Attached gingival<br>Alveolar ridge<br>Dorsum of tongue<br>Tonsils<br>Pillars of fauces<br>Uvula		Score = total of sites affected<br>1 for each site in group 1 (non-keratinised mucosa)<br>2 for each site in group 2 (keratinised, specialised, or oropharynx)

Evidence of scarring Yes ☐ No ☐

Total OUSS=

**Quality of Life**<br>(on a scale of 0-10)<br>as perceived by patient	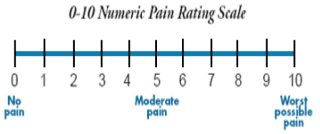	Maximum Score=10<br>i.e.1 =slight discomfort when ulcers are present<br>i.e. 10= excruciating ulcers interfering with eating, talking and smiling.
Eating		
Talking		
Smiling		

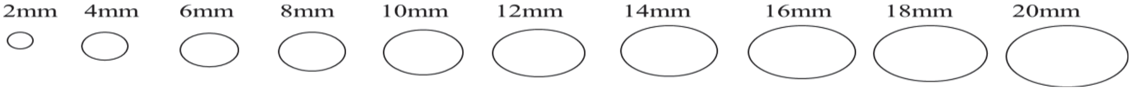

Table S2: Periodontal risk factors summary in BD and RAS patients

Periodontal risk factors	Number BD Pt.	%	Number RAS Pt.	%
**Gingival history**	57	39%	7	35%
**Family gingival history**	37	25%	4	20%
**plaque retention factors (supra-gingival calculus, sub-gingival calculus, poor restoration margins)**	111	76%	16	80%
**Smoking**				
• **Never smoked**	83	56%	13	65%
• **Smokers**	43	29.4%	3	15%
• **Smoke cessation**	20	13.7%	3	15%
**Systemic diseases**				
• **Pt. only with BD**	70	48%	8	40%
• **Psychological & social wellbeing**	54	37%	7	35%
• **Diabetes**	6	4%	2	10%
• **Cardiovascular problems**	7	4.8%	1	5%
• **Others**	22	15%	2	10%
**Diet**				
•**Balanced**	126	86%	16	80%
**Oral hygiene habits**				
• **Teeth brushing**				
➢ **Once**	22	15%	4	20%
➢ **Twice**	100	68%	14	70%
➢ **Three times**	16	11%	2	10%
➢ **No**	7	4.8%	0	0
• **Teeth flossing**				
➢ **Regular flossing**	49	34%	7	35%
➢ **Irregular flossing**	87	60%	11	55%
➢ **No**	8	5.4%	2	10%
• **Tongue brushing**				
➢ **Regular**	48	33%	9	45%
➢ **Irregular**	93	64%	7	35%
➢ **No**	4	3%	4	20%

Table S3: Oral ulceration management guideline in BD

Oral problems	Treatment	Considerations
**Oral ulcer**	**First line; less complex disease**<br>1-Oral colchicine 500 mcg twice/day<br>2-Topical steroid (mouthwash, gel, spray)<br>3-Topical non-steroidal mouthwash (anti-inflammatory mouthwash)<br>4-Barts mouthwash↑<br>5-Antibiotics<br><br>**Second line; for the 30% ineffective group**<br>1-Azathioprine 2mg/Kg (up to dose 200 mg/day) usually started at low dose before increasing<br>OR<br>2-Mycophenolate (up to 3g/day)<br>OR/AND<br>3-Steroid (orally, IM, IV)<br>Variable daily, weekly or monthly<br><br>**Third line; TNF inhibitor for the 33% ineffective group**<br>1-Infliximab 5mg/Kg doses then consider swapping to subcutaneous TNFi after 4 weeks.<br>(stop therapy after 6 months, and restart if flare)	Effective in 70%<br>↓<br>Ineffective in 30%<br><br>Effective in 66%<br>↓<br>Ineffective in 33%
**Dental caries**	1-Good oral hygiene<br>(Brushing teeth at least twice/daily, free Sodium Lauryl Sulphate (SLS) toothpaste, and flossing daily)<br>2-Decrease sugary foods/drinks<br>3-Regular dental examination	
**Periodontitis**	1-Same steps for dental caries control<br>2-Smoking cessation	
**Candidiasis**	1-Same steps for dental caries/periodontitis control<br>2-Disinfect removable prosthesis each night<br>3-Topical antifungal therapy<br>4-Systemic antifungal therapy	
**Scarring**	1-Topical steroid therapy<br>2-Patients with stenosis are referred for dilation.	

†Barts Mouthwash (1 tablet Betamethasone 500 mcg + 1 tablet Doxycycline 100 mg + 1 ml Nystatin 100.000 unit/ml dissolved in 10 ml of water) and rinsing up to 3 minutes.

Figure S1: Dental caries risk and basic periodontal examination results in BD and RAS patients

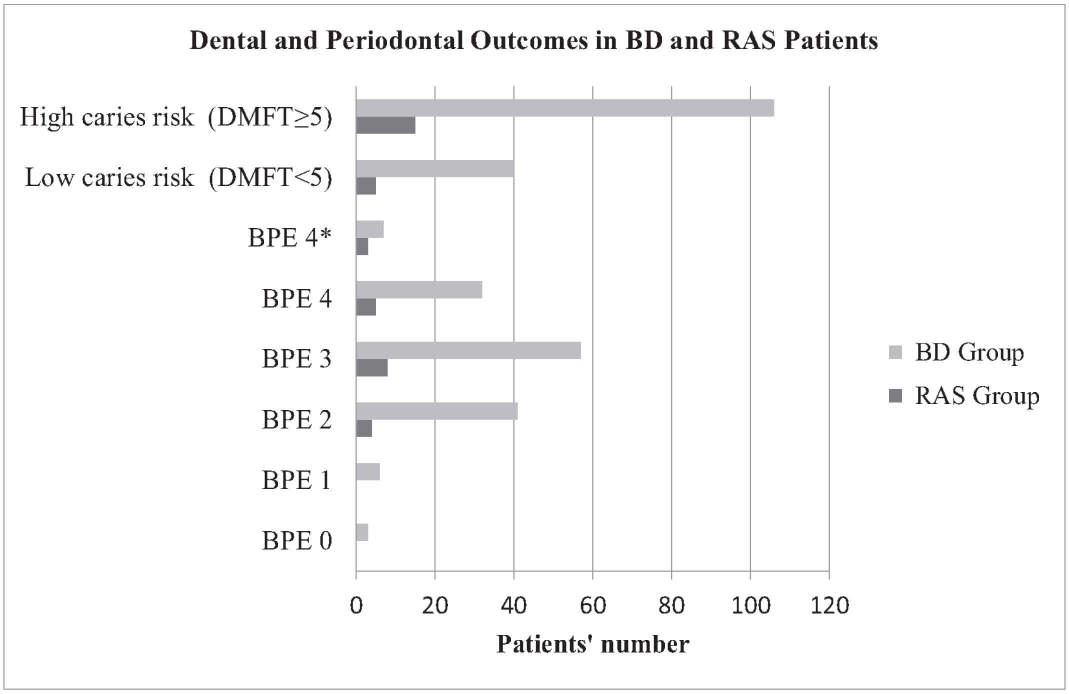

Supplement: Supplementary file 1 — Supplementary material 1 (PDF 235 KB) [file 296_2018_4117_MOESM1_ESM.pdf]
